# Cell Wall Integrity Signaling in Fruit Ripening

**DOI:** 10.3390/ijms24044054

**Published:** 2023-02-17

**Authors:** Kenan Jia, Wei Wang, Qing Zhang, Wensuo Jia

**Affiliations:** 1 College of International Education, Beijing University of Chemical Technology, Beijing 100029, China; 2 College of Horticulture, China Agricultural University, Beijing 100193, China; 3 Key Laboratory for Agricultural Application and New Technique, College of Plant Science and Technology, Beijing University of Agriculture, Beijing 102206, China; 4 Institute of Horticulture Crops, Xinjiang Academy of Agricultural Science, Xinjiang, Urumqi 830000, China

**Keywords:** cell wall integrity, FERONIA, fruit ripening, THESEUS

## Abstract

Plant cell walls are essential structures for plant growth and development as well as plant adaptation to environmental stresses. Thus, plants have evolved signaling mechanisms to monitor the changes in the cell wall structure, triggering compensatory changes to sustain cell wall integrity (CWI). CWI signaling can be initiated in response to environmental and developmental signals. However, while environmental stress-associated CWI signaling has been extensively studied and reviewed, less attention has been paid to CWI signaling in relation to plant growth and development under normal conditions. Fleshy fruit development and ripening is a unique process in which dramatic alternations occur in cell wall architecture. Emerging evidence suggests that CWI signaling plays a pivotal role in fruit ripening. In this review, we summarize and discuss the CWI signaling in relation to fruit ripening, which will include cell wall fragment signaling, calcium signaling, and NO signaling, as well as Receptor-Like Protein Kinase (RLKs) signaling with an emphasis on the signaling of FERONIA and THESEUS, two members of RLKs that may act as potential CWI sensors in the modulation of hormonal signal origination and transduction in fruit development and ripening.

## 1. Introduction

As each plant cell is tightly connected to its neighbors through shared walls, plant tissues form a continuum of cells [1]. Thus, plant growth and morphogenesis are mechanistically controlled by the properties of cell walls. Plants have evolved signaling mechanisms to monitor the changes in the cell wall structure, thereby triggering corresponsive responses to sustain cell wall integrity (CWI) [2,3,4]. Changes in cell wall architecture can occur under environmental stresses or some developmental processes [5]. However, while CWI signaling in relation to environmental stresses has been extensively studied, less attention has been paid to the development-associated CWI signaling. Fruit ripening is a complex process involving dramatic changes in numerous biochemical metabolisms, such as color, sugar, acid, flavor, firmness, etc., among which the decrease in fruit firmness resulting from cell wall modification has been increasingly suggested to be the most important event that is tightly coupled with fruit ripening [5,6]. The pattern of cell wall modification during fruit ripening is unique in that it is a programmed and irreversible process, such that the cell walls may be nearly totally weakened at the late stage of fruit ripening [6]. Thus, a question has been raised about whether cell wall modification during fruit ripening may be linked to CWI signaling and whether CWI signaling may act to control fruit ripening.

Receptor-like kinases (RLKs) are serine/threonine kinases with extracellular domains to perceive the cell wall-associated signals [7,8]. Accordingly, it has been proposed to play an important role in CWI signaling. In the model plant, *Arabidopsis,* RLKs constitute a superfamily that contains more than 600 members, which can be again categorized into 12 subfamilies [1,8], such as Catharanthus roseus RECEPTOR-LIKE KINASE1-LIKE Proteins (CrRLK1Ls), Wall-Associated Kinases (WAKs), Leucine-Rich Repeat Receptor-Like Kinases and Receptor-Like Protein (LRR-RLKs), etc. Among these RLKs, the CrRLK1Ls family, which consists of 17 members, has conceived special attention owing to their critical roles in the diversity of biological processes [7,9,10,11,12,13,14,15]. Nevertheless, most of the of CrRLK1Ls have not been functionally characterized except for FER1 and THE1, two members of CrRLK1Ls, which have increasingly demonstrated to be key sensors of CWI impairment [16,17,18,19,20,21,22,23]. In particular, emerging evidence suggests that FER and THE may play important roles in fruit ripening via modulation of hormone biosynthesis and signal transduction [24,25,26,27].

Besides RLKs, there exist two other groups of signals that may be tightly coupled with CWI signaling. One group derives from cell wall metabolites. For example, cellulose and the pectin metabolism-associated oligo-saccharide fragments have been demonstrated to be capable of triggering cellular signaling in response to CWI impairment caused by environmental stresses [28,29,30,31,32,33,34]. Another is the calcium (Ca^2+^) signal, which has been well established to play critical roles in a diversity of biological processes. Plant cell walls have been regarded as extracellular Ca^2+^ pools, where Ca^2+^ is largely bound to pectin [35,36,37]. As fruit ripening is accompanied by pectin degradation, it is reasonable to propose that the content of free Ca^2+^ would substantially change along with fruit growth and development. Consequently, Ca^2+^ signaling should play an important role in fruit ripening. However, studies and reviews on the wall modification associated-Ca^2+^ signaling have been lacking.

Hormones, such as ethylene and abscisic acid (ABA), are the main determinants of fruit ripening. According to the physiological features of fruit ripening, fleshy fruits can be categorized into two major groups, i.e., climacteric fruits (CL) that show dramatic increases in respiration and ethylene production during fruit ripening, and non-climacteric fruits (NC) that do not show a typical increase in respiration and ethylene production [38,39]. It has been suggested that NC fruit ripening is controlled by a synergistic action of abscisic acid (ABA), auxin (IAA), brassinosteroid (BR) [40,41,42], and jasmonic acid (JA) [43,44,45]. To date, our knowledge about the mechanisms for hormonal signal production and transduction in fruit ripening is still limited. As CWI signaling has been increasingly suggested to play pivotal roles in the modulation of both hormone biosynthesis and signal transduction, mechanistic investigations of the ripening-associated CWI signaling would contribute to a profound understanding of the hormonal signaling in fruit development and ripening. In the past decades, studies on CWI signaling have been extensively reviewed. However, these reviews have mainly focused on CWI signaling in response to environmental stresses [1,3,8]. This review aims to bring together recent advances in our knowledge of CWI signaling linked to fruit ripening. We highlight the mechanisms for the roles of FER and THE, the two extensively studied CWI sensors, in the early hormonal signal origination and downstream signal production in fruit development and ripening.

## 2. Cell Wall Modification, along with Fruit Development and Ripening

### 2.1. Architecture

Fruit softening is the key event in fruit ripening. Fruit ripening is a highly coordinated and genetically programmed process involving a series of structural, physiological, and biochemical changes, finally leading to the development of edible ripe fruit with desirable quality attributes [46]. Softening is a phenomenon unexceptionally occurring during fruit ripening. Although the pattern of softening varies greatly depending on species and cultivars, e.g., some fruits, such as apricot, papaya, and peach, soften greatly and become a melting texture, whereas some others, such as apple, pear, and jujube, may soften moderately and become a crisp texture, fleshy fruits all become seriously modified as they fully ripen [6,47].

Fruit softening has been thought to result from cell wall modification. Besides, it has also been suggested to be affected by cell turgor [48,49]. In the past years, cell wall modification in fruit ripening has been extensively reviewed [5,46,47,50,51,52,53,54,55,56,57]. Here, we briefly summarize the mechanistic aspects of cell wall modification, highlighting the essential role of cell wall modification in the initiation of fruit ripening.

The plant cell wall is a complex composite of polysaccharides and proteins, which can be again categorized into several broad classes, including cellulose, hemicellulose, pectins, expansins, etc. [5,58,59]. The procedure of cell wall modification has been proposed to commonly start with cell expansion and separation and involve concerted and synergistic action of suites of enzymes, where one family of cell wall modifying proteins might mediate the activity of another, thus leading to orchestrating cell wall degradation [60,61]. However, less is known about how the concerted and synergistic action of these enzymes is conducted. In a recent study on strawberry fruit ripening [6], we observed that the cell wall degradation is likely undertaken in a spatially ordered manner, i.e., starts with the middle lamina separation, then gradually disassembling from the middle area towards the inside. Therefore, cell separation is the key event controlling the overall cell wall degradation. As the middle lamina is mainly composed of pectin, this observation is consistent with the fact that pectin degradation has been well-established to play a major role in fruit softening [39,62]. In addition to cell wall degradation, it has been proposed that a decrease in cell turgor might be an important factor in promoting fruit softening. A study by Wada et al. [49] reported that the ripening of grape (*Vitis vinifera*) was accompanied by an apoplastic solute accumulation, thus resulting in a dramatic decrease in cell turgor. ‘Delayed Fruit Deterioration’(DFD) is a tomato cultivar exhibiting dramatically delayed softening [48] and, therefore, remarkably delayed fruit deterioration. The firm feature of DFD was demonstrated to result from much-reduced water loss because DFD shows several unusual features of cuticle composition and architecture, indicating that the change in cell turgor can be an important part of fruit softening [48].

As it has long been known that fruit ripening is essentially regulated by hormonal signals, the change in cell metabolism during fruit ripening has been generally thought to be a result of hormonal signaling. In other words, the change in cell wall metabolism has been merely regarded as an event initiated by fruit ripening [5]. However, based on the theory of CWI signaling, a change in cell wall architecture would expect to initiate cellular signaling whereby initiating cell wall modification to sustain cell wall integrity. Since fruit ripening is unavoidably accompanied by a dramatic change in cell wall architecture, it is reasonable to propose that the ripening-associated changes in cell wall architecture may potentially act to trigger signaling events to promote fruit ripening, which implies that the cell wall metabolic is essentially linked with other ripening-associated metabolisms, such as color, sugar, acid, aroma, texture, etc. Such a point of view can be strongly supported by an early study on a pleiotropic dominant mutation of *Solanum lycopersicum*, named a colorless non-ripening mutation (*Cnr*) [63,64]. *Cnr* has a phenotype characterized by the fruit that shows inhibition of softening, yellow skin, and a non-pigmented pericarp [64]. Further analyses indicate that the *Cnr* phenotype is tightly coupled with a feature of much reduced cell-to-cell adhesion, principally owing to an enhanced cell separation in the fruit pericarp [65]. This strongly suggests that cell wall separation may play a key role in the regulation of overall fruit ripening. Based on an anatomical study of fruit ripening in strawberries (*Fragaria vesca*), we observed that cell separation occurred from the very earliest stage of the fruit set. Moreover, the time course of the accelerated cell separation just coincided with the onset of ripening, implying an important role of cell separation in the initiation of strawberry fruit ripening [6].

While decades of research have attempted to prevent fruit softening through a delay of the entire ripening process [39,47,50,58,66], less attention has been paid to the fact that regulation of softening may interfere with other ripening aspects, such as color, sugar, flavor, and nutrient, etc. Actually, the main goal of improving fruit quality is to uncouple softening from other fruit quality-associated events rather than merely delay the process of fruit ripening. Because of this, it is quite important to understand whether/how fruit softening is tightly coupled with other ripening aspects. Studies on cell wall medication in relation to fruit ripening may contribute to profoundly understanding the coupling mechanisms of fruit ripening-associated metabolisms. A putative mechanism is that cell wall modification may function in the activation of a series of signaling cascades whereby regulating fruit ripening and quality formation, which will be discussed in detail in the following sections.

### 2.2. Molecular Basis of Cell Wall Modification

The fruit cell wall is principally composed of cellulose, hemicelluloses, pectin, and a small fraction of proteins [60]. It has been generally accepted that cellulose microfibrils are coated with and crosslinked together with hemicellulose and with filled in the spaces of the cellulose network [50,51]. The hemicellulose, which may contribute to cell rigidity, is mainly made up of xyloglucans, xylans, and glucomannans. Pectins, which are a diverse group of heteropolysaccharides containing galacturonic acid residues, are the common and major components of the primary cell wall and middle lamella and have an important role in cell-to-cell adhesion [60,61,62]. There exist three major classes of pectins: homogalacturonan (HG), rhamnogalacturonan 1 (RG-I), and rhamnogalacturonan II (RG-II). Expansins are a class of cell wall-localized proteins and have been increasingly suggested to be key regulators of wall loosening and extension during growth. Several lines of evidence indicate that expansins may play an important role in the regulation of fruit firmness and ripening [52,53,54].

Cell wall modification in ripening fruit is highly complex, involving diverse families of wall-modifying proteins [51]. Among these proteins, the enzymes involved in pectin degradation have been generally thought to play a major role in the regulation of fruit firmness [47]. There are many studies attempting to promote fruit firmness by inhibiting the gene expression of PG, a key enzyme of pectin degradation. In transgenic tomatoes, however, 99% inhibition of the PG activity in ripening fruit was not found to have a significant effect on fruit firmness [67], implying that PG is not the sole contributing factor toward fruit softening. Subsequent research on other pectin metabolic enzymes, such as pectin methylesterase (PME) [68,69] and galactanase (b-Gase) [70], were not able to demonstrate a strong effect on fruit softening. A recent study by Paniagua et al. (2020) reported that antisense suppression of two PG-encoding genes, *FaPG1* or *FaPG2*, induced the expression of a putative high-affinity potassium transporter. As the potassium transporter contributes to the promotion of cell turgor, it was proposed that the PGs’ modification of cell wall might be linked to the loss of cell turgor, which was again proposed to be the basis for the regulation of fruit firmness [66]. Logically, soluble solid accumulation during fruit ripening would expect to cause a decrease in osmotic potential, whereby an increase rather than a decrease in cell turgor. The question lies in that cell wall weakening contributes to the release of the wall constraint, thereby reducing cell turgor. Although cell turgor can be a factor affecting fruit firmness, the reduction of fruit firmness during fruit ripening may be more likely a comprehensive result of the changes in wall architecture, cell expansion, and separation.

To date, there are many attempts to modify softening through overexpression or silencing of genes encoding cell wall modifying proteins [58,71,72], including expansin [73], but this research only observed modest changes in firmness. It is not surprising to find the lack of success in the regulation of fruit firmness via manipulation of a single gene, owing to the complexity of cell wall composition. For example, pectin is composited of a diverse group of hetero-polysaccharides. Thus degradation of each group of pectin requires a variety of enzymes, and each enzyme is encoded by a number of genes. Additionally, dissemble the cell walls is a highly ordered process, as described above, such that cell walls can be effectively dismantled only in an orderly manner.

## 3. CWI Signal Candidates in Fruit Ripening

### 3.1. Cell Wall Fragment Signals

In the past decades, studies on CWI signaling have been mainly focusing on plant immunity. Pathogen-associated molecular patterns (PAMPs) and damage-associated molecular patterns (DAMPs) have been identified to be primary signals to trigger plant immunity responses [32,74,75,76,77,78,79]. Some DAMPs essentially belong to cell wall metabolites, among which oligogalacturonides (OGs) have conceived considerable attention owing to their important roles in plant immunity responses. There is evidence that OGs signaling may also be involved in the regulation of plant growth and development. For example, an early study by Simpson et al. [80] reported that oligomers across a range of polymerization from 4–6 derived from polygalacturonase-digested citrus pectin were able to trigger ethylene production and fruit ripening in a tomato plant, and a study by Branca et al. [81] reported that OGs prepared by partial hydrolysis of sodium polypectate was reported to be capable of inhibiting the auxin-induced elongation of pea (*Pisum sativum*) stem. Similarly, OGs were shown to inhibit auxin-induced root formation in *tobacco* and *Arabidopsis* leaf explants [82,83]. In strawberries, Osorio et al. [84] reported that ectopic expression of *FaPE1*, a gene encoding pectin methyl esterase from *F. ananassa*, decreased the size of OGs extracted from the ripe *F. vesca* fruits, and application of the OGs from the transgenic fruits more strongly induced the expression of PR5, a maker gene of pathogen resistance, compared to that from the wild type. Moreover, ectopic expression of *FaPE1* promoted the content of aromatic amino acids phenylalanine, tryptophan, tyrosine, and aspartate. More recently, Yang et al. [85] reported that OGs prepared from apple pectin through enzymatic digestion using a mixture of PG and PL from *Botrytis cinerea* inhibited fruit softening in ripening tomatoes. This research suggest that OGs are indeed capable of serving as signals to regulate plant growth and development. However, to date, studies on changes in endogenous OGs in fruit growth and ripening have been lacking. Accordingly, it can’t currently be concluded that OGs are key signals implicated in fruit ripening.

### 3.2. Ca^2+^ Signaling

Ca^2+^ is well known as a second messenger that plays powerful roles in diverse biological processes. One of the bases for Ca^2+^ to act as a signal is its compartmentalized distribution with cell walls as a pool where the content of Ca^2+^ may be ten thousand times higher than the cytoplasm, such that, in responses to stimuli, Ca^2+^ can quickly enter into cells to initiate downstream signaling [35,36,37]. In cell walls, Ca^2+^ is largely bound to pectin [86,87]. Consequently, pectin degradation would expect to cause an elevation of free Ca^2+^, although there is a report that the total content of the extracellular Ca^2+^ was not significantly altered during fruit ripening [86]. Pharmacological studies reported that the application of Ca^2+^ on fruit surface is capable of significantly affecting fruit ripening [88,89,90], implying that an increase in free Ca^2+^ in the apoplast, as a result of pectin degradation, would expect to have an impact on fruit ripening.

In support of pharmacological studies, molecular research has increasingly suggested that many Ca^2+^ signaling-associated components, such as the Ca^2+^ sensor, calmodulin, Ca^2+^-dependent protein kinases, and CDPK, might be implicated in fruit ripening [91,92,93,94,95]. In tomatoes, for example, it was reported that the gene expression of many Ca^2+^/calmodulin-regulated SR/CAMTAs was dramatically altered in ripening mutant *rin* compared with wildtype fruit, and moreover, transiently overexpressing *SlCaM2* in mature green fruit delayed ripening, while reducing *SlCaM2* expression accelerated ripening, implying involvement of Ca^2+^/Calmodulin-associated signaling in the regulation of fruit development and ripening [93]. A number of studies indicate that Ca^2+^ signaling acts to interact with hormonal signaling, jointly regulating fruit ripening [87,96,97]. There is no doubt that Ca^2+^ signaling plays an important role in the regulation of fruit ripening. But from the point of view of CWI signaling, it remains unclear whether the cell wall modification may alter the content of extracellular free Ca^2+^ and whether this alteration may contribute to the regulation of fruit ripening. To conclusively demonstrate that Ca^2+^ signaling is involved in fruit ripening, investigations on the change of extracellular free Ca^2+^ along with fruit development and ripening and its effect on fruit ripening are required.

### 3.3. NO Signaling

Nitric oxide (NO) has been well established to play a crucial role in plant growth and environmental adaptation [98,99]. As a free radical, NO could also react with various intracellular/extracellular targets, thus forming a series of NO derivatives termed reactive nitrogen species (RNS) [100], such as NO radicals (NO−), nitrosonium ions (NO+), peroxynitrite (ONOO−), S-nitrosothiols (SNOs), etc. In animals, NO biosynthesis is well characterized to be from l-arginine by NO synthases (NOSs) [101]. In plants, NO generation is thought to occur at mitochondria and chloroplasts are organelles [102,103], and in addition to the non-enzymatic reduction of nitrite, several enzymes, such as unknown protein (s) with a NOS-like activity, nitrate reductase (NR), and nitrite: NO reductase, are believed to be responsible for NO generation [104,105]. NO may function synergistically or antagonistically with ROS in many biological processes [106,107]. The roles of NO signaling in plant development and environmental adaptions have been extensively reviewed. As this review focuses on CWI in fruit ripening, this part only summarized and discussed the NO signaling in fruit ripening with emphasis on its linkage to CWI signaling.

A pioneering study by Leshem et al. [108] reported the involvement of NO signaling in fruit ripening. Subsequent studies suggest that NO plays an important role in fruit ripening. Nevertheless, most of the studies are based on pharmacological approaches [109,110,111]. For example, the application of NO was reported to be capable of delaying fruit ripening in tomato [110,111], strawberry, and kiwi fruit [112], Papaya (*Carica papaya* L. *cv. ‘Sui you 2′*) [113] or even capable of extending the shelf life of vegetables, such as broccoli (*Brassica oleracea*), green bean (*Phaseolus vulgaris*) [114], pepper (*Capsicum annuum* L.) [109]. In support of the pharmacological evidence, a study by Bodanapu et al. [115] demonstrated that a tomato shr mutant resulted in NO accumulation and suppressed fruit growth and ripening. In addition, it has been increasingly suggested that NO signaling plays pivotal roles in the modulation of both hormone biosyntheses and signal transduction, such as abscisic acid (ABA), salicylic acid, and jasmonic acid [116,117]. Importantly, it has been established that NO functions in suppression of the activities of ACS and ACO, two key enzymes in the ethylene biosynthesis pathway, implying that NO signaling should play key roles in the regulation of fruit ripening [118,119,120].

As mentioned above, OGs play important roles in CWI signaling. There is evidence that OGs were capable of triggering NO production in many biological processes. For example, a study by Hu et al. [121] (2003) reported that OGs stimulated NO accumulation in the growth medium of Panax ginseng through activation of NOS, and a study by Lecourieux et al. [122] (2005) reported that OGs stimulated NO accumulation in tobacco cells. In *Arabidopsis*, Rasul et al. [123] (2012) demonstrated that OGs triggered NO production, and moreover, the NO accumulation acted to modulate RBOHD-mediated ROS production and participated in the regulation of OG-responsive genes such as anionic peroxidase (*PER4*) and a β-1,3-glucanase. Importantly, an S-Nitroso-Proteome study by Navarro et al. (2020) demonstrated that, in response to nitrosative stress, a large number of proteins were S-nitrosylated, among which several proteins are involved in CWI signaling, such as Hsp60 and Hsp90 as well as the Arp2/3 complex subunit that has been suggested to regulate chitin and β-glucans metabolism thereby affecting CWI in Candida albicans [124,125]. Overall, these studies strongly suggest that there is a linkage between NO and CWI signaling. Interestingly, a more recent study by Duan et al. [126] (2022) demonstrated that pollen tube arrival at the ovule induced NO accumulation at the filiform apparatus, and the NO accumulation was FER-dependent, as evidenced by the findings that the *fer-4* mutant lacked comparable activity of NO accumulation. These observations provide direct support for a signaling linkage between FER and NO.

### 3.4. CrRLK1Ls Signaling in Fruit Ripening

Plant receptor-like protein kinases (RLKs) belong to an evolutionarily conserved superfamily with more than 600 members in *Arabidopsis* [127,128]. Their extracellular ligand-binding domain (ECD) is extensively diversified [129]. The RLKs superfamily can be divided into 12 subfamilies based on their ECD structures [130]. Among these subfamilies, the Catharanthus roseus receptor-like kinase 1-like proteins (CrRLK1Ls) is a subfamily that has been studied extensively and well established to play pivotal roles in numerous biological processes. The CrRLK1Ls family is named after the first isolated member, CrRLK1, from Madagascar periwinkle [131]. Members of the CrRLK1L subfamily share a malectin-like domain (MLD). In the plant model *Arabidopsis*, the CrRLK1L subfamily contains 17 members, many of which have been characterized, e.g., THESEUS1 (THE1), HERCULES1 (HERK1), HERCULES2 (HERK2), FERONIA/SIRENE (FER/SIR), ANXUR1 (ANX1), ANXUR2 (ANX2), etc. and among these characterized members, FER and THE have attracted particular interest, owing to their special roles in CWI signaling [14,132,133]. CrRLK1Ls have been reviewed many times [7,10,14,15,23]. As this article aims to review CWI signaling in fruit ripening, we will summarize and discuss our recent knowledge on the roles and mechanisms of FER and THE signaling in fruit ripening, with other members and biological processes not covered in the present review.

#### 3.4.1. FER Signaling in Fruit Ripening

##### Role of FER in Fruit Ripening

FER was named for the Etruscan goddess of fertility due to its important role in reproduction [134]. The mechanism for FER to control fertility lies in that FER acts to control pollen tube growth and rupture, whereby controlling the release of sperms into a gametophyte. In the *Arabidopsis* mutant *feronia* (*fer*), the pollen tube fails to arrest and thus continues to grow inside the female gametophyte without rupture for the release of sperms [17,18]. Besides the role in reproduction, accumulating evidence suggests that FER plays critical roles in a diversity of biological processes, such as root growth [135,136,137], immune responses [21,138,139,140,141,142], environmental adaptations [143,144,145,146], etc.

FER contains malectin-like domains that have been demonstrated to be capable of interacting with cell-wall polysaccharides, such as pectin, which led to the speculation that it can potentially function in CWI signaling. Because cell wall degradation is a key event accompanied by fruit ripening, it is reasonable to propose that FER may likely play a critical role in fruit ripening. In accordance with this proposal, we previously demonstrated that FER plays an important role in fruit ripening [24,25,26]. In apples, for example, overexpression and antisense expression of *MdFERL6* in apple fruit callus inhibited and promoted ethylene production, respectively. By using virus-induced gene silencing (VIGS), it was found that down-regulation of *SlFERL1*, a homolog gene of *MdFERL6*, promoted tomato fruit ripening. Moreover, MdFERL6 and MdFERL1 could physically interact with MdSAMS (S-adenosylmethionine synthase), a key enzyme in the ethylene biosynthesis pathway, implying that FER may regulate apple and tomato fruit ripening vial modulation of ethylene production. In strawberries *(Fragaria × ananassa*), we found that overexpression and RNAi-mediated downregulation of *FaMRLK47*, a member of FER, delayed and accelerated fruit ripening, respectively. FaMRLK47 could physically interact with FaABI1, a negative regulator of abscisic acid (ABA) signaling, implying that FER plays an important role in the regulation of strawberry fruit ripening [24]. More recently, a study by Ji et al. [27] reported that the MADS-box transcription factor RIPENING-INHIBITOR (RIN), one of the major factors controlling tomato fruit ripening, could directly bind to the promoter of *SlFERL* (FERNOIA-like) to activate *SlFERL* expression. Importantly, overexpression of *SlFERL* significantly accelerated the ripening process of tomato fruit, whereas RNA interference knockdown of *SlFERL* delayed fruit ripening. Although, to date, there is scarce research on FER signaling in relation to fruit ripening, the existing evidence conclusively demonstrates that FER should be a key signal controlling both CL and NC fruit ripening.

As discussed above, Ca^2+^, NO, and ROS are potential signals implicated in the regulation of fruit ripening. Strikingly, it has been increasingly suggested that FER signaling may act to trigger the release of these signals. For example, a study by Gjetting SK et al. [147] (2020) reported that RALF, the ligand of FER, could trigger signaling in *Arabidopsis,* and more recently, a study by Gao et al. [148] (2022) demonstrated that FER signaling acts to trigger Ca^2+^ signaling for pollen tube reception. Also, a study by Luo et al. [141] (2022) reported that FER signaling could mediate calcium ion homeostasis implicated in the immune response. Importantly, Duan et al. [126] (2020) demonstrated that FER is crucial for maintaining de-esterified pectin at the filiform apparatus, and pollen tube arrival at the ovule triggered the accumulation of nitric oxide at the filiform apparatus, whereby suppressing pollen tube attraction. Also, there is evidence that FER signaling acts trigger ROS production in a variety of biological processes [149,150,151]. These observations imply that FER signaling should be tightly coupled with fruit ripening.

##### FER Modulation of Hormone Signaling

- ABA signaling

Hormones are well known to be the major determinant of fruit ripening, among which ethylene acts to control CL fruit ripening, whereas a synergistic action of ABA, IAA, JA, and BR act to control NC fruit ripening [38,39,43,44,45]. Emerging evidence suggests that FER acts to modulate the signal transduction of nearly all hormones. The best studied is the FER modulation of ABA signaling. In *Arabidopsis*, FER signaling is initiated by an interaction of the FER kinase with guanine exchange factors, such as GEF1, GEF4, and GEF10, which subsequently activates GTPase ROP11/ARAC10. A study by Yu et al. [152] reported that *Arabidopsis* mutants disrupted in any step of the FER signaling pathway displayed an ABA hypersensitive response. Further studies demonstrate that the ROP11/ARAC10 protein physically interacted with the ABI2 phosphatase and enhanced its activity. Intriguingly, besides ROP11/ARAC10, a subsequent study by Chen et al. [153] demonstrated that FERONIA could directly interact with ABI2-type phosphatases and be dephosphorylated by ABI2. As ABI2 is a negative regulator of ABA signaling, the dephosphorylation of FER by ABI2 acts to activate FER signaling. Moreover, the ABA-dependent FER activation required the involvement of the ABA receptor, PYRABACTIN RESISTANCE (PYR)/PYR1-LIKE (PYL)/REGULATORY COMPONENTS OF ABA RECEPTORS (RCAR). As mentioned above, in strawberries, a FER homolog FaMRLK47 was demonstrated to be capable of physically interacting with FaABI1. Overall, these studies suggest that the FER and ABA signaling are actually reciprocally modulated, implying a tight correlation between the two pathways.

- IAA signaling

IAA is a major regulator of cell growth [5]. Studies suggest that, besides plant growth, IAA also plays a pivotal role in NC fruit ripening [154,155,156,157,158,159,160]. In strawberries, for example, it has been well established that IAA serves as a key signal negatively controlling fruit ripening. Specifically, IAA acts to promote fruit growth and expansion, and a dramatic decrease in the IAA level contributes to the initiation of fruit ripening [154]. With regards to the mechanism for IAA to regulate cell growth, it has been traditionally thought that auxin acts to trigger the activation of plasma membrane-localized H+-ATPases, resulting in acidification of the intercellular space [161]. Meanwhile, there is evidence that exogenous auxin application may trigger apoplast alkalization in roots, which is the opposite effect as in shoots [162,163]. A study by Barbez et al. [162] demonstrated that IAA-induced apoplast alkalinization is dependent on FER, as evidenced by the observation that the *fer-4* mutant seedlings displayed a substantial resistance to apoplast alkalization. *fer-4* mutant displays increased lateral root branching and delayed gravitropic response, which is associated with polar auxin transport (PAT). Moreover, the *fer-4* mutant resulted in asymmetric root growth, which can be prevented by suppressing the polar auxin transport in *fer-4* [164,165]. These observations suggest that FER and auxin signaling function in a synergistic regulation of the wall pH and growth.

- BR signaling

BR receptor, BRI1, belongs to receptor-like kinase BRASSINOSTEROID INSENSITIVE 1. Upon BR ligand binding, BRI1 and its co-receptor BAK1/somatic embryogenesis receptor kinase (SERK)3 transphosphorylation each other, initiating a signaling cascade leading to the regulation of transcription factors BZR1 and BES1/BZR2 [166]. A number of studies suggest that inference with the activity of pectin methylesterase (PME), a key enzyme controlling cell wall homeostasis, activated BR signaling. Interestingly, activation of BR signaling regulated the expression of many cell wall biosynthesis and remodeling genes, including several PMEs, thus forming a feedback regulation of BR signaling to ensure homeostasis of cell wall biosynthesis and remodeling, thereby protecting plants against CWI impairment caused by the imbalance in pectin modification [167,168,169]. These observations reveal that BR signaling is an integrated part of CWI signaling. The importance of BR signaling in cell wall modification implies its essential connection to CrRLK1L kinase signaling, which has been proposed to be a negative regulator of cell wall stiffness [19]. In accord with the suspected link between BR and CrRLK1L kinase signaling, there are studies that report that the expression of *FER* as well as two other tightly linked genes, *HERCULES1* and *THESEUS1*, are target genes of BR signaling, as evidenced by those observations that BZR1 could bound to the promoters of FERONIA2 (*FER2*) and *FER3* whereby directly inducing their expression, and moreover, *fer* knockout mutant shows altered responsiveness to BR [170]. Overall, these studies suggest that FER is a key modulator of BR signaling. Nevertheless, little is known about whether the effect of BR on fruit ripening is linked with FER signaling.

- JA signaling

JA signaling pathway consists of COI1, JAZ, and MYC2. COI1 was identified as the JA receptor, which is essentially a ubiquitin ligase, targeting the JASMONATE ZIM-domain (JAZ) repressor for degradation by the 26S proteasome [171]. As a transcription factor, MYC2 is a master regulator of the JA signaling pathway. It orchestrates the JA pathway by controlling the gene expression of the JA responses in numerous biological processes, such as plant response to pathogen infection, abiotic stress adaptation, and growth and development [171]. JA signaling has been established to play a critical role in fruit ripening [39,154]. Emerging evidence suggests that JA signaling may be linked with FER signaling, jointly regulating plant immunity. For example, Guo et al. [172] reported that FER might act to inhibit JA and COR signaling by phosphorylating and destabilizing MYC2, but intriguingly, the peptide ligand RALF23 may act through FER to stabilize MYC2 and elevate JA signaling. In addition, Zhao et al. [173] reported that the cell wall leucine-rich repeat extensins LRX3/4/5, RALF22/23, and FER function as a module, negatively regulating JA biosynthesis. The *lrx345* triple mutant displays many similar phenotypes with the *fer-4* mutant, suggesting that FER signaling may be linked to the functional regulation of extensins. In the *lrx345* triple mutant, many JA and SA-responsive genes are constitutively up-regulated, whereas disruption of the JA pathway, via crossing *lrx345* to the coi1-1 mutant, suppressed the dwarf phenotype of the *lrx345* and *fer-4* mutants, implying the functional of extensin by FER is correlated to JA signaling. More recently, Darwish et al. [174] reported that FER was a strong negative regulator of JA-dependent touch signaling, as evidenced by the observations that the expressional pattern of a wide range of touch-responsive genes for JA biosynthesis was dramatically altered in the *fer-4* mutant.

- Ethylene signaling

A decade ago, a screen for *Arabidopsis* mutants with increased ethylene response revealed a transferred DNA (T-DNA) insertion in the coding sequence of *FER*, suggesting an important role of FER in ethylene signaling [170]. A subsequent study by Mao et al. [175] demonstrated that FER could physically interact with two S-adenosylmethionine synthases, a key enzyme in the ethylene biosynthesis pathway. *fer* mutants led to a dwarf phenotype with an elevated level of SAM and ethylene. Jia et al. [25] reported that heterologous expression of *MdFERL6* or *MdFERL1*, the apple homolog of *Arabidopsis FER*, fruit delayed ripening, and suppressed ethylene production. Virus-induced gene silencing (VIGS) of *SlFERL1*, the tomato homolog of *FER*, promoted tomato fruit ripening and ethylene production. Both MdFERL6 and MdFERL1 were found to be capable of physically interacting with S-adenosylmethionine synthase, further demonstrating the role of *FER* in the regulation of fruit ripening via modulation of ethylene production [25]. RIPENING INHIBITOR (RIN) and AGAMOUS-LIKE1 (TAGL1) are key regulators of fruit ripening. More recently, a study by Ji et al. [27] reported that RIN and TAGL1 could directly bind to the promoter region of *SlFERL* and activate its expression. Thus, *FER* and Ethylene signaling may constitute a loop of modification, i.e., while *FER* acts to modulate ethylene biosynthesis, ethylene signaling functions to regulate *FER* expression, thereby promoting *FER* signaling.

#### 3.4.2. THE Signaling in Fruit Ripening

THE1 was discovered in a screen for suppressors that attenuated the short hypocotyl phenotype of dark-grown *Arabidopsis* in the mutation background of cellulose synthase *CESA6* [176]. As a relative of FER, it shares similar signaling mechanisms with FER, as evidenced by the observations that it could interact with RALF34 and GUANINE EXCHANGE FACTOR4 (GEF4), which have been respectively identified to be the ligand and the downstream signaling component of the FER kinase [13,177,178]. Studies on THE1 have focused on its role in CWI signaling. Isoxaben (ISX) and Driselase are chemicals that are commonly used to study CWI-associated signaling because they are respectively capable of affecting cellulose biosynthesis or showing a similar effect of the enzyme cocktail released by fungal pathogens during infection. A study by Engelsdorf et al. [75] reported that JA accumulation could be induced by Driselase treatment. Moreover, the Driselase-induced JA accumulation was respectively reduced in *the1-1* (i.e., a mutant of loss of function) and enhanced in *the1-4* (i.e., a mutant of gain of function) seedlings upon treatment with active Driselase compared to Col-0 seedlings, suggesting that THE1 was implicated in the CWI impairment-induced JA accumulation [75]. Very recently, Gigli-Bisceglia et al. [179] reported that salt stress triggered PME activation, and the double mutants of *herk1* and *the1-4* strongly responded to salt stresses, implying that THE1 plays a role in stress response via regulating PME metabolism. These observations demonstrate that THE1 can be a key sensor of CWI.

Although much less is known about the role of THE in hormonal signaling compared to that of FER, a number of studies suggest that THE1 indeed plays pivotal roles in both hormonal biosynthesis and signal transduction. For example, a study by Guo et al. [180] showed that THE1, as well as its relative, HERK, could be induced by BR, and moreover, the *herk1 the1* double mutant partially suppressed the cell elongation phenotype of *bes1-D*, a gain of function mutant that displays constitutive BR responses, suggesting that HERK1 and THE1 cooperate with BR pathway and mediate part of BR-regulated cell elongation. In addition, Nikonorova et al. [181] reported that NAA treatment induced phosphorylation of THE1 at Ser668 and *ralf34-1* mutant, a ligand of THE1, displayed reduced sensitivity to NAA in its growth-suppressing responses, implying that THE1 is implicated in the modulation of IAA signaling. More importantly, a recent study by Bacete et al. [22] reported that THE1 could modulate both ABA and JA biosynthesis, as evidenced by the findings that JA accumulation was induced by ISX treatment. Furthermore, the JA accumulation was respectively reduced and enhanced in the *the1-1* (i.e., loss of function) and *the1-4* (i.e., gain of function) mutant seedlings compared to Col-0 controls. Meanwhile, it was found that the sorbitol-induced ABA accumulation was enhanced in *the1-4* seedlings compared to Col-0 controls, suggesting that THE1 acted as a negative regulator of ABA biosynthesis. The mechanism for dehydration-induced ABA accumulation has long been a key and intriguing research topic for plant scientists, but so far, little is known about it. The findings that THE acts to mediate ABA biosynthesis is a breakthrough in the research on the ABA signal production in response to developmental and environmental stimuli. As both ABA and JA are key regulators of fruit ripening, it is of great significance to explore whether THE signaling is implicated in fruit ripening.

## 4. Conclusions and Perspective

**Figure 1:**
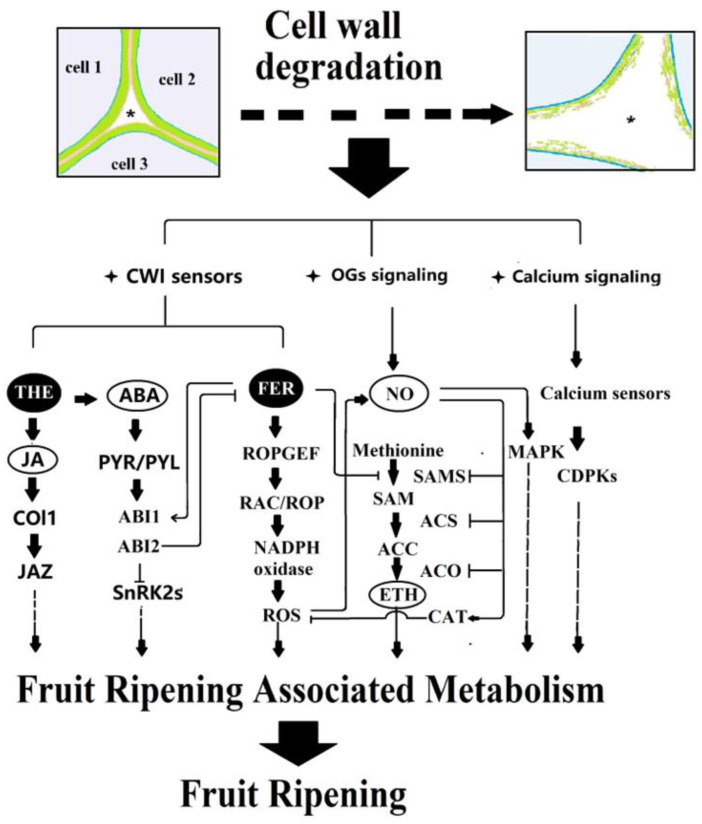
Proposed diagram showing CWI signaling in fruit ripening. Fruit growth and development are accompanied by cell wall degradation, which starts from cell separation resulting from pectin degradation. The cell wall degradation triggers several signaling systems, including RLK-associated signaling, OGs signaling and Ca^2+^ signaling, NO signaling, etc. Among the RLK-associated signals, CrRLK1Ls, particularly FER, and THE, have been established to play pivotal roles in both hormone signal production and transduction. OGs and Ca^2+^ signaling may potentially play important roles in the initiation of fruit ripening via or not via modulation of hormone signaling. NO potentially acts as a signal downstream of OGs signals and is proposed to be linked with multiple signaling pathways, among which the ethylene biosynthesis pathway has been well characterized. *, apoplastic space of tricells, showing cell separation and wall degradation.The wall model is based on a study by Zhang et al. [6] RLK, Receptor Like Protein kinase; OGs, oligosaccharides; FER, Feronia; THE, Theseus. SnRK2, sucrose nonfermenting 1-related protein kinase 2, PYR/PYL, ABA receptor; ABI1/2, protein phosphatase 2C; RopGEF, guanine nucleotide exchange factors; RAC/ROP, small GTPase; ROS, reactive oxygen species; CAT, catalase; NO, nitric oxide; SAMS, S-adenosyl-L-methionine synthetase; ACC, 1-aminocyclopropane-1-carboxylic acid; ACS, ACC synthetase; ACO, ACC oxidase; ETH, ethylene; JA, jasmonic acid; COI1, coronatine-insensitive 1; JAZ, jasmonate ZIM-domain; MAPK, mitogen-activated protein kinase; CDPK, Ca^2+^ dependent protein kinase.

Figure 1 is a proposed diagram showing the CWI signaling in relation to fruit ripening. In summary, fruit growth and development are accompanied by cell wall degradation, which starts from cell separation resulting from pectin degradation. Cell wall degradation is proposed to trigger several signaling systems, among which RLK-associated signaling, Ca^2+^ signaling, and OGs signaling potentially play pivotal roles in the initiation of fruit ripening. NO potentially acts as a signal downstream of OGs and FER and has been established to modulate ethylene biosynthesis. Hormones, especially ethylene and ABA, are well-known internal cues controlling fruit ripening. Acting as important sensors of CWI, FER, and THE have been increasingly demonstrated to be capable of modulating hormone biosynthesis and signal transduction. To further unravel the molecular mechanism for the control of fruit ripening, it is of particular significance and importance to explore the following aspects:

### 4.1. Ca^2+^ Signaling in Relation to Cell Wall Degradation during Fruit Ripening

Ca^2+^ signaling has been well demonstrated to play important roles in fruit ripening, but it is largely unknown how Ca^2+^ signaling is initiated. As cell walls serve as an extracellular pool of Ca^2+^, degradation of the walls would expect to cause a change in the level of free Ca^2+^, thereby producing an impact on Ca^2+^ signaling. Investigation of the changing pattern of free Ca^2+^ as affected by cell wall degradation would contribute to a profound understanding of the signaling mechanisms for the regulation of fruit ripening.

### 4.2. CWI Signaling in Relation to Hormonal Signal Production

Hormones are well known to be key regulators of fruit ripening. However, little is known about the mechanisms for their origination, along with fruit development and ripening. In *Arabidopsis*, FER and THE have been suggested to play an important role in the biosynthesis of multiple hormones, such as ethylene, ABA, and JA. Further studies are required to demonstrate whether FER and THE may function in hormonal production during fruit ripening.

### 4.3. Reciprocal Modulation between Hormonal and CWI Signaling

As CWI sensors, FER and THE have been increasingly suggested to play important roles in modulating multiple hormone signaling via cross-talk between CWI and hormonal signaling. Conversely, many hormones have been suggested to be capable of modulating FER and THE-associated signaling via regulating the expressions of FER and THE. Thus there exists a reciprocal modulation between hormone and CWI signaling, reflecting the complexity of the mechanisms for the regulation of fruit ripening. Investigation of the mechanisms for the reciprocal modulation between hormone and CWI signaling would contribute to a better deciphering of the signaling networking controlling fruit ripening.

## Data Availability

Not applicable.
